# *KNSTRN*, a Poor Prognostic Biomarker, Affects the Tumor Immune Microenvironment and Immunotherapy Outcomes in Pan-Cancer

**DOI:** 10.1155/2023/6729717

**Published:** 2023-02-15

**Authors:** Wanli Zhang, Yanxia Liao, Chengdong Liu, Li Liu, Xiaohan Zhou

**Affiliations:** ^1^Department of Infectious Diseases, Nanfang Hospital, Southern Medical University, Guangzhou, Guangdong, China; ^2^Department of Radiation Oncology, Nanfang Hospital, Southern Medical University, Guangzhou, Guangdong, China

## Abstract

Kinetochore-localized astrin- (SPAG5-) binding protein (*KNSTRN*) is mainly involved in mitosis. Somatic mutations in *KNSTRN* are known to lead to the occurrence and development of certain tumors. However, the role of *KNSTRN* in the tumor immune microenvironment (TIME) as a tumor prognostic biomarker and potential therapeutic target has not been clarified. Accordingly, in this study, we aimed to investigate the role of *KNSTRN* in the TIME. mRNA expression, cancer patient prognosis, and correlations between *KNSTRN* expression and immune component infiltration were analyzed using Genotype-Tissue Expression, The Cancer Genome Atlas, Cancer Cell Line Encyclopedia, Human Protein Atlas, ImmuCellAI, TIMER2.0, and KM-Plotter. The Genomics of Drug Sensitivity in Cancer database was used to evaluate the relationship between *KNSTRN* expression and the half maximal inhibitory concentration (IC50) of several anticancer drugs, and gene set variation analysis was performed. Data were visualized using R version 4.1.1. *KNSTRN* expression was upregulated in the majority of cancers and was associated with a worse prognosis. Additionally, *KNSTRN* expression was highly correlated with the infiltration of multiple immune components in the TIME and was related to a poor prognosis in tumor patients receiving immunotherapy. *KNSTRN* expression was also positively correlated with the IC50 of various anticancer drugs. In conclusion, *KNSTRN* may be a significant prognostic biomarker and promising target for oncotherapy in numerous cancers.

## 1. Introduction

Malignant tumors, which pose a serious threat to human health, require more effective treatments. New targets must be identified, and novel drugs need to be developed. Because the tumor immune microenvironment (TIME) affects the occurrence and development of cancer in intricate ways, it has attracted increasing attention from the medical community. The TIME features a wide variety of immune cells and contains noncellular components such as cell-surface molecules, immune checkpoints, and soluble immune factors [1]. During tumorigenesis, tumor cells first achieve immune evasion. Next, the process of tumor expansion begins, which directly or indirectly affects the secretion of cytokines and the infiltration and function of immune cells in the TIME. Finally, the tumor grows and metastasizes [1]. The classifications and numbers of immune cells in the TIME vary according to the type of tumor, as well as among different patients with the same tumor type. Among the immune cells in the TIME, natural killer (NK) cells, cytotoxic T lymphocytes, and antigen-presenting cells act as tumor suppressor cells, whereas regulatory T cells (Tregs), tumor-associated macrophages, and myeloid-derived suppressor cells promote immunosuppression [2]. In order to improve cancer treatments, the discovery and verification of new biomarkers in the TIME are necessary.

Kinetochore-localized astrin- (SPAG5-) binding protein (*KNSTRN*) encodes a centromere-associated protein involved in chromosome division [3]. *KNSTRN* is mutated under ultraviolet irradiation and has attracted attention as an oncogene in cutaneous squamous cell carcinoma (SCC), the second most common skin cancer. *KNSTRN* has also been reported to play a role in melanoma. Together with *TP53* and *CDKN2A*, *KNSTRN* is one of the three most commonly mutated genes [4]. *KNSTRN* is also frequently somatically mutated in basal cell carcinoma in addition to cutaneous SCC and melanoma, although this mutation usually occurs in advanced stages of the tumor [5]. Somatic mutations in *KNSTRN* not only cause the occurrence of malignant tumors but also promote tumor development. *KNSTRN* promotes the movement of AKT to PIP3 and stimulates AKT phosphorylation, resulting in metastasis and gemcitabine resistance in bladder cancer [6]. Studies on *KNSTRN* have mainly focused on somatic mutations, and research on the involvement of *KNSTRN* in tumor immunity remains scarce, even though *KNSTRN* has been shown to have a significant influence on immune cells in the tumor microenvironment of lung adenocarcinoma (LUAD) [7]. Additionally, *KNSTRN* has been mainly researched in individual tumors; pan-cancer studies on *KNSTRN* are lacking.

Therefore, to better understand the expression of *KNSTRN*, as well as the associations between *KNSTRN* expression and patient prognosis, immune score, tumor-associated immune cells, immune-related components, and the half maximal inhibitory concentration (IC50) of anticancer drugs, we conducted a series of bioinformatics analyses. We aimed to determine whether *KNSTRN* may serve as a potential pan-cancer prognostic biomarker and to explore the influence of *KNSTRN* on the TIME and anticancer therapy outcomes.

## 2. Materials and Methods

### 2.1. Data Collection

RNA expression and clinical data from the Genotype-Tissue Expression (GTEx) database and The Cancer Genome Atlas (TCGA) were obtained from the UCSC Xena database (https://xenabrowser.net/datapages/). The expression of *KNSTRN* in tumor cell lines was evaluated using the Cancer Cell Line Encyclopedia (CCLE) (https://sites.broadinstitute.org/ccle). The tissue immunohistochemical (IHC) and immunofluorescence (IF) images of various tumor cells were obtained from the human Protein Atlas (HPA) database (https://www.proteinatlas.org/).

### 2.2. Prognostic Analysis

Univariate Cox regression analyses were performed to estimate the significance of *KNSTRN* expression in predicting pan-cancer overall survival (OS), disease-specific survival (DSS), disease-free interval (DFI), and progression-free interval (PFI). Kaplan-Meier analysis was performed to evaluate the OS, DSS, PFI, and DFI of patients from TCGA cohort. OS and progression-free survival (PFS) data of cancer patients receiving immunotherapy were obtained from the Gene Expression Omnibus database.

### 2.3. Immune Cell Infiltration

We obtained and evaluated the immune cell infiltration score of TCGA cohort from the ImmuCellAI database (http://bioinfo.life.hust.edu.cn/web/ImmuCellAI/), TIMER2 database (http://timer.cistrome.org/), and published studies [8, 9]. Immune score is the level of all immune cells in the tumor microenvironment calculated using the R package “estimate.” Similarly, infiltration score is the infiltration level of the total immune cells in the tumor microenvironment calculated by ImmuCellAI database (http://bioinfo.life.hust.edu.cn/ImmuCellAI#!/). All cancer patients were divided into two groups (high or low *KNSTRN* expression) based on the median *KNSTRN* expression level to determine the influence of *KNSTRN* expression on the extent of immune cell infiltration.

### 2.4. Correlation Analysis and Gene Set Variation Analysis (GVSA)

Correlation analysis of the expression of *KNSTRN* and all related genes was performed using TCGA pan-cancer data, and the Pearson correlation coefficient was calculated. GSVA was conducted using the R package “GSVA” to calculate the pathway score of each sample, based on MSigDB v7.1 (https://www.gsea-msigdb.org/gsea/msigdb/index.jsp) with the following parameters: nPerm = 1000, minGSSize = 10, maxGSSize = 1000, and *p* value cutoff = 0.05.

### 2.5. Drug Response Analysis

The IC50 values of 192 drugs and gene expression profiles of 809 cell lines were obtained from the Genomics of Drug Sensitivity in Cancer database (GDSC) (https://www.cancerrxgene.org/). Correlation analysis was conducted to identify associations between *KNSTRN* expression and IC50 value.

### 2.6. Statistical Analyses

Data are presented as the mean ± standard error. Differences between groups were analyzed using Student's *t-*test or analysis of variance. Statistical analyses were performed using R version 4.1.1. Results were considered statistically significant at *p* < 0.05 (two-tailed).

## 3. Results

### 3.1. Expression of *KNSTRN* in Pan-Cancer Data

To establish *KNSTRN* expression profiles from pan-cancer data, expression analysis of *KNSTRN* was conducted in 33 types of tumors and 31 types of paired normal tissues, using data from TCGA database and the GTEx database. *KNSTRN* expression in tumor tissues was higher than that in normal tissues in the majority of tumors, including the following 27 tumor types: adrenocortical carcinoma (ACC), bladder urothelial carcinoma (BLCA), breast invasive carcinoma (BRCA), cervical squamous cell carcinoma and endocervical adenocarcinoma (CESC), cholangiocarcinoma (CHOL), colon adenocarcinoma (COAD), lymphoid neoplasm diffuse large B-cell lymphoma (DLBC), esophageal carcinoma (ESCA), glioblastoma multiforme (GBM), head and neck squamous cell carcinoma (HNSC), kidney chromophobe (KICH), kidney renal clear cell carcinoma (KIRC), kidney renal papillary cell carcinoma (KIRP), brain lower grade glioma (LGG), liver hepatocellular carcinoma (LIHC), lung adenocarcinoma (LUAD), lung squamous cell carcinoma (LUSC), ovarian serous cystadenocarcinoma (OV), pancreatic adenocarcinoma (PAAD), prostate adenocarcinoma (PRAD), rectum adenocarcinoma (READ), sarcoma (SARC), skin cutaneous melanoma (SKCM), stomach adenocarcinoma (STAD), thymoma (THYM), uterine corpus endometrial carcinoma (UCEC), and uterine carcinosarcoma (UCS).

**Figure 1:**
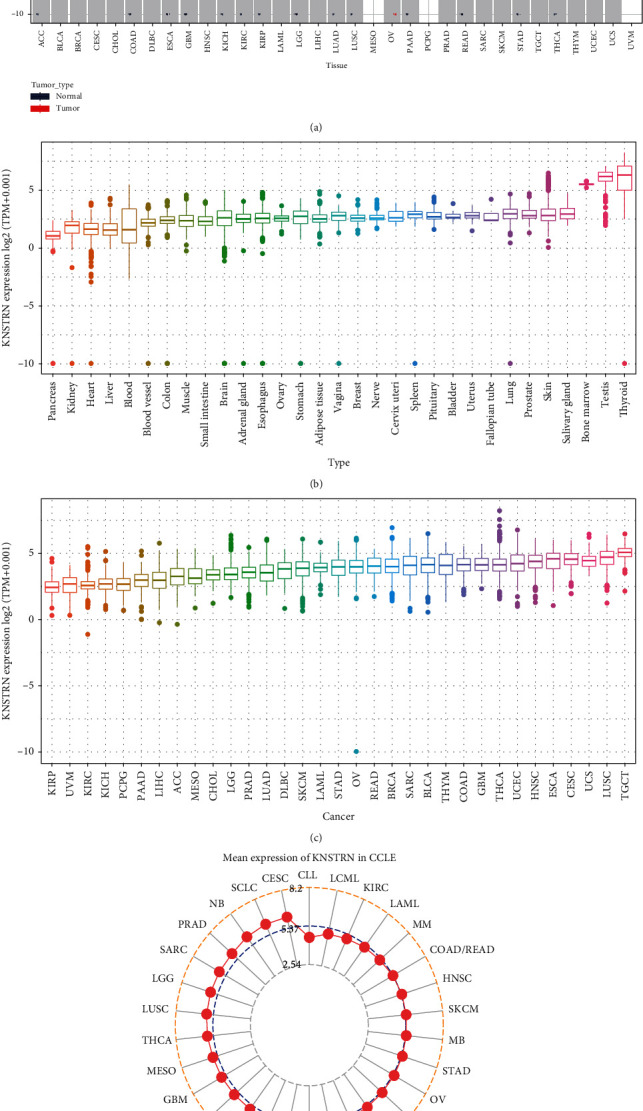
Pan-cancer mRNA expression of *KNSTRN*. (a) Analysis of the mRNA expression of *KNSTRN* in tumor tissues using TCGA database and in normal tissues using the GTEx and TCGA databases. (b) The mRNA expression of *KNSTRN* in normal tissue from the GTEx database. (c) The mRNA expression of *KNSTRN* in tumor tissue from TCGA database. (d) The mRNA expression of *KNSTRN* in tumor cell lines from the CCLE database.

In contrast, there were three tumors—acute myeloid leukemia (LAML), testicular germ cell tumors (TGCT), and thyroid carcinoma (THCA) —in which *KNSTRN* was downregulated in tumor tissues (Figure 1(a)). Expression analysis of *KNSTRN* in normal tissues indicated that *KNSTRN* expression was the highest in the thyroid and the lowest in the pancreas (Figure 1(b)). Among cancer tissues, *KNSTRN* expression was the highest in TGCT and the lowest in KIRP (Figure 1(c)). Furthermore, among cancer cell lines, the expression of *KNSTRN* was highest in CESC cells and lowest in CLL cells (Figure 1(d)).

**Figure 2:**
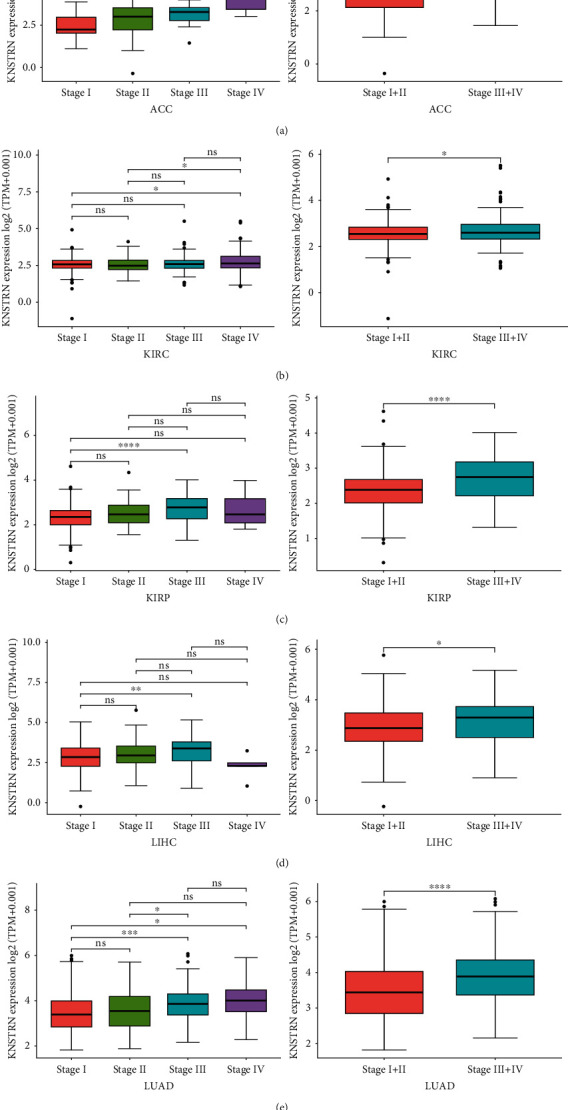
Correlation between the expression of *KNSTRN* and TNM tumor stages. (a–e) Determination of pan-cancer *KNSTRN* expression in four different TNM stages from TCGA database. The left panel represents stage I-IV and the right panel merges stage I with stage II, and merges stage III with stage IV. ^∗^*p* < 0.05;  ^∗∗^*p* < 0.01;  ^∗∗∗^*p* < 0.001, and^∗∗∗∗^*p* < 0.0001; ns: not significant.

**Figure 3:**
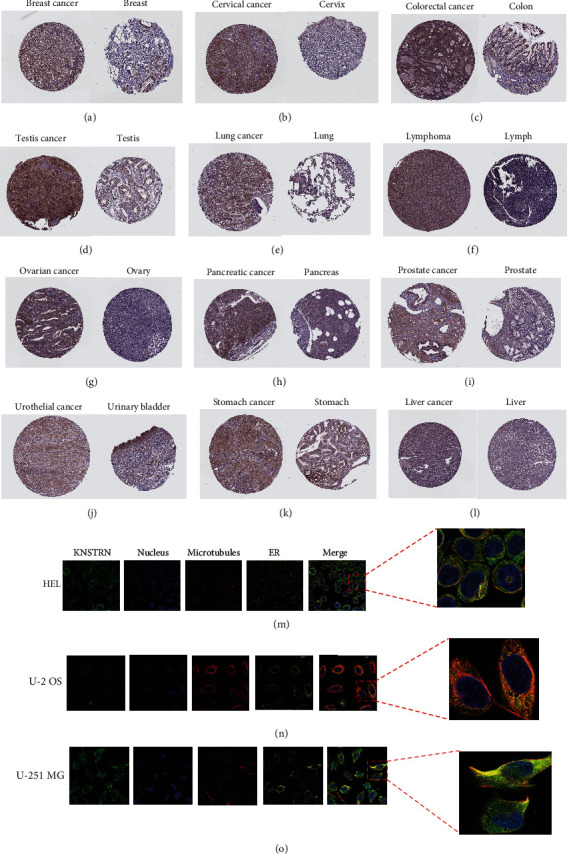
Pan-cancer analysis of *KNSTRN* expression in tumor cells. (a–l) Immunohistochemical analysis of *KNSTRN* in tissues of breast cancer, cervical cancer, colorectal cancer, testis cancer, lung cancer, lymphoma, ovarian cancer, pancreatic cancer, prostate cancer, urothelial cancer, stomach cancer, liver cancer, and corresponding normal tissues. (m–o) The expression of *KNSTRN* in acute erythroid leukemia cell (HEL), osteosarcoma cell (U-2 OS), and astrocytoma cell (U-251 MG) by using Immunofluorescence assay.

To further characterize the expression of *KNSTRN*, we analyzed *KNSTRN* expression in tumors and paired adjacent tissues. In 27 types of tumors, *KNSTRN* expression in tumor tissues was upregulated compared to that in paired adjacent tissues **(**Figure S1A-L). Next, we assessed the expression of *KNSTRN* in four different TNM stages. *KNSTRN* was highly expressed in the following five types of tumors at advanced stages (stage III/IV): ACC, KIRC, KIRP, LIHC, and LUAD (Figures 2(a)–2(e)). In addition, we used the HPA database to assess IHC data and found that breast cancer (breast invasive ductal carcinoma), cervical cancer (cervical squamous cell carcinoma), colorectal cancer (CRC) (colorectal squamous cell carcinoma), testis cancer (seminoma), lung cancer (lung adenocarcinoma), lymphoma (non-Hodgkin lymphoma), ovarian cancer (ovarian endometrioid carcinoma), pancreatic cancer (pancreatic adenocarcinoma), prostate cancer (prostate adenocarcinoma), bladder cancer (urothelial cancer), stomach cancer (gastric adenocarcinoma), and liver cancer (hepatocellular carcinoma) tissues exhibited higher *KNSTRN* expression than the corresponding normal tissues (Figures 3(a)–3(l)). We then used immunofluorescence to observe *KNSTRN* expression at the cellular level in three different tumor cells: acute erythroid leukemia cell (HEL), osteosarcoma cell (U-2 OS), and astrocytoma cell (U-251 MG) (Figures 3(m)–3(o)).

### 3.2. *KNSTRN* Expression Influences the Prognosis of Cancer Patients

**Figure 4:**
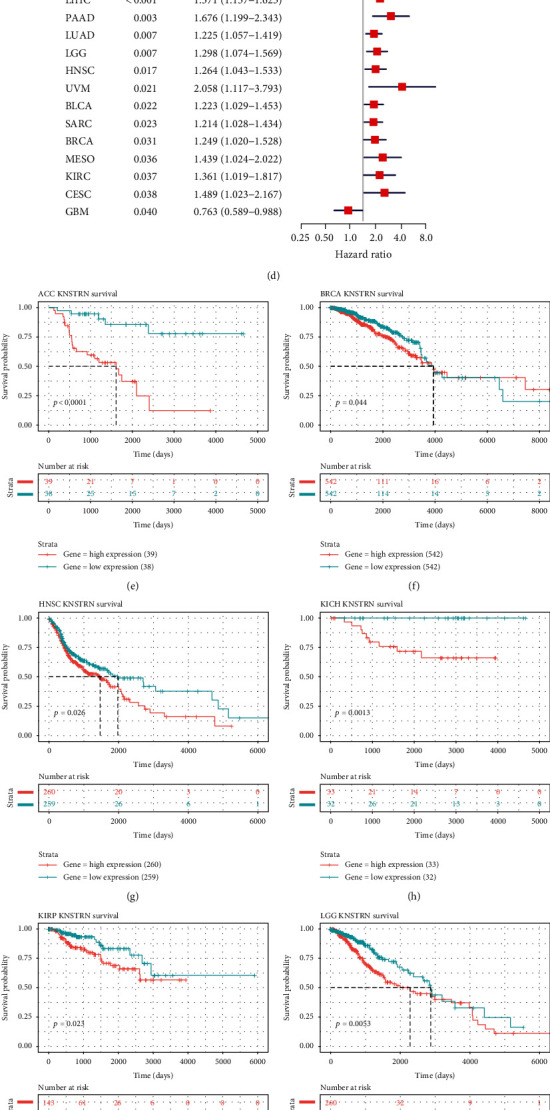
Univariate Cox regression analysis and survival analysis of *KNSTRN*. (a–d) The forest map displays the pan-cancer univariate Cox regression outcomes of *KNSTRN* for overall survival (OS), disease-free interval (DFI), disease-specific survival (DSS), and progression-free interval (PFI) in The Cancer Genome Atlas (TCGA). (e–o) Kaplan-Meier analysis of the correlation between the OS of cancer patients and *KNSTRN* expression by using TCGA database. The median value of *KNSTRN* expression in each tumor was taken as the cut-off value.

To fully understand the effect of *KNSTRN* expression on cancer prognosis, we analyzed the survival outcomes of patients with cancer in the TCGA cohort. The results of univariate Cox regression analysis indicated that *KNSTRN* was a risk factor for OS in LGG, KIRP, ACC, KICH, LUAD, MESO, PAAD, LIHC, PCPG, KIRC, HNSC, BRCA, and BLCA. In contrast, *KNSTRN* was a protective factor in THYM (Figure 4(a)). *KNSTRN* was also a risk factor for DFI in KIRP, LIHC, PAAD, BRCA, SARC, CESC, and ACC (Figure 4(b)); for DSS in KIRP, LGG, KICH, ACC, LUAD, MESO, PAAD, KIRC, LIHC, PCPG, HNSC, PRAD, and BRCA (Figure 4(c)); and for PFI in ACC, KIRP, KICH, LIHC, PAAD, LUAD, LGG, HNSC, UVM, BLCA, SARC, BRCA, MESO, KIRC, CESC, and GBM (Figure 4(d)).

Furthermore, Kaplan-Meier analysis of the effect of *KNSTRN* on OS, DSS, PFI, and DFI indicated similar results (Figures 4(e)–4(o), Figure S2A-Y). Overall, high expression of *KNSTRN* in most tumors led to poor prognosis, confirming that *KNSTRN* is a tumor risk factor. Its specific role in tumors should be examined to facilitate follow-up intervention.

### 3.3. *KNSTRN* Expression Is Closely Associated with the TIME

**Figure 5:**
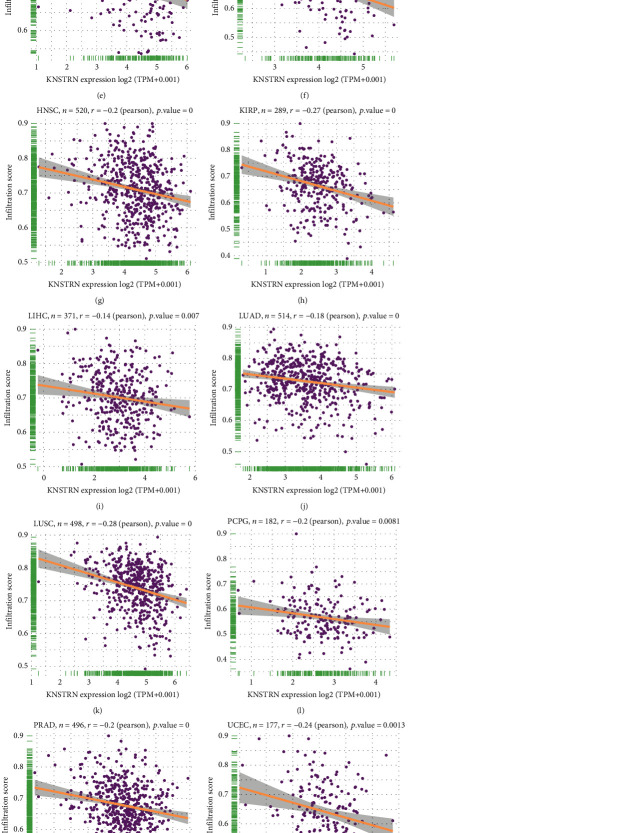
Association of immune infiltration score with *KNSTRN* expression in a variety of cancers. (a) Correlation between *KNSTRN* and immune score in pan-cancer. The location of the dot represents the mean value of *KNSTRN* expression. (b–t) Correlation between *KNSTRN* and infiltration score in ACC, CESC, CHOL, ESCA, GBM, HNSC, KIRP, LIHC, LUAD, LUSC, PCPG, PRAD, UCEC, SARC, SKCM, TGCT, THYM, THCA, and UVM.

**Figure 6:**
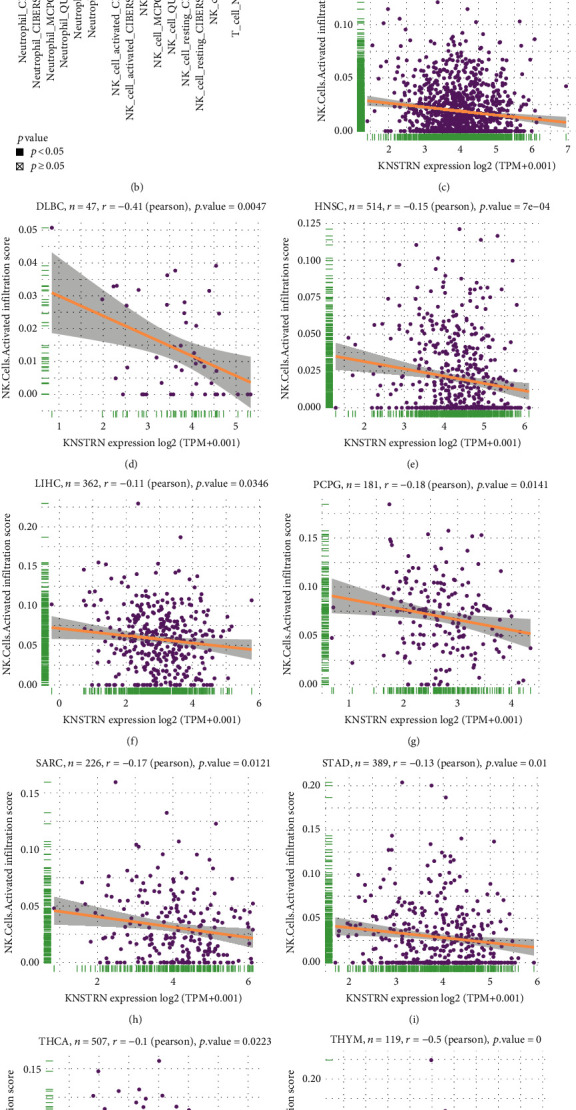
*KNSTRN* is related to immune cell infiltration. (a) Relationship of *KNSTRN* expression with immune cell infiltration using the ImmuCellAI database. (b) Relationship of *KNSTRN* expression with immune cell infiltration using the TIMER2 database. (c–p) Association of *KNSTRN* expression with NK cell in various cancers from a published work. ^∗^*p* < 0.05,  ^∗∗^*p* < 0.01,  ^∗∗∗^*p* < 0.001, and ^∗∗∗∗^*p* < 0.0001.

Immune cells and molecules are crucial components of the immune microenvironment and are closely related to its state. The immune cell infiltration score reflects the activity of the TIME. We first examined the immune score (Figure 5(a)) and infiltration score (Figures 5(b)–5(t)) in pan-cancer data; *KNSTRN* expression was correlated with tumor immune suppression. Then, we conducted a correlation analysis to evaluate the relationship between *KNSTRN* expression and immune cell infiltration, using the ImmuCellAI database, TIMER database, and published data. According to the results (Figures 6(a) and 6(b)), *KNSTRN* expression was positively associated with the infiltration of neutrophils, Tregs, macrophages, and NK resting cells, but negatively associated with that of NK activated cells, CD8^+^ T lymphocytes, and CD4^+^ T lymphocytes in multiple tumors. These findings were corroborated by published research (Figures 6(c)–6(p), Figure S3A-S).

**Figure 7:**
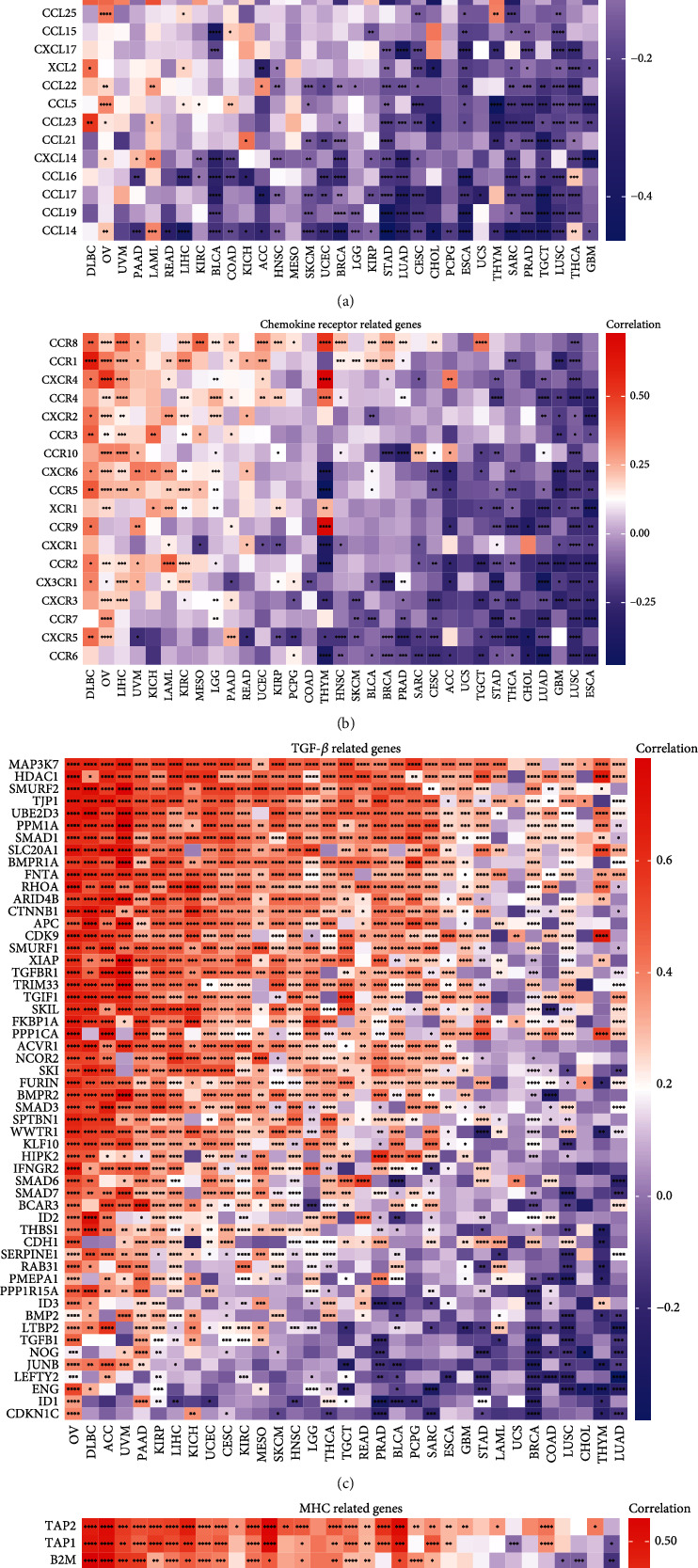
*KNSTRN* is associated with immune progress-related genes. (a) Correlation between *KNSTRN* and chemokine-related genes using TCGA database. (b) Correlation between *KNSTRN* and chemokine receptor-related genes using TCGA database. (c) Correlation between *KNSTRN* and TGF-*β* pathway-related genes using TCGA database. (d) Correlation between *KNSTRN* and MHC-related genes using TCGA database. ^∗^*p* < 0.05,  ^∗∗^*p* < 0.01,  ^∗∗∗^*p* < 0.001, and^∗∗∗∗^*p* < 0.0001.

**Figure 8:**
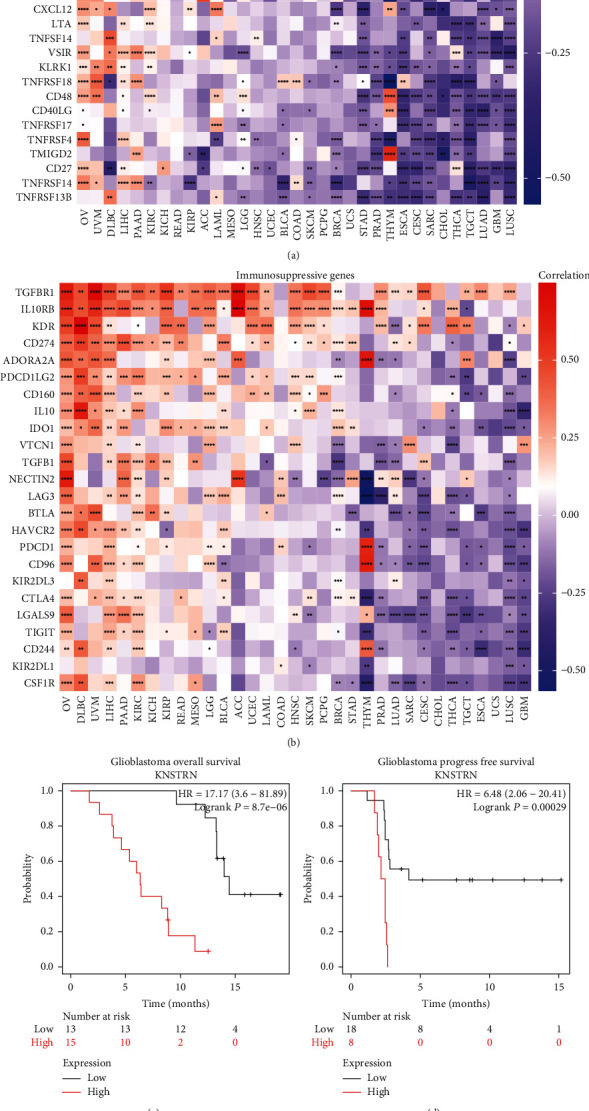
*KNSTRN* is associated with immune activity-related genes and the prognosis of tumor patients with immunotherapy. (a) Evaluation of the correlation of *KNSTRN* expression with immune activating genes using TCGA database. (b) Correlation of *KNSTRN* expression with immunosuppressive genes using TCGA database. (c–e) Correlation of *KNSTRN* expression with the prognosis of some tumor patients receiving immunotherapy using Kaplan-meier Plotter database. ^∗^*p* < 0.05,  ^∗∗^*p* < 0.01,  ^∗∗∗^*p* < 0.001, and ^∗∗∗∗^*p* < 0.0001.

In addition to immune cells, immune molecules have a significant influence on the TIME. We analyzed the correlation between *KNSTRN* expression and that of chemokine-related genes (Figure 7(a)), chemokine receptor-related genes (Figure 7(b)), and TGF-*β* pathway-related genes (Figure 7(c)), which possess immunosuppressive functions. We also evaluated the expression of major histocompatibility complex-related (MHC) genes (Figure 7(d)), which are associated with immunobiological processes. All of the above gene types were strongly associated with *KNSTRN* in pan-cancer data. Moreover, we identified a significant correlation between *KNSTRN* expression and that of immunosuppressive and immune-activating genes (Figures 8(a) and 8(b)). Since *KNSTRN* was significantly associated with immune cells and molecules in the TIME, we further evaluated the relationship between *KNSTRN* and the prognosis of cancer patients receiving immunotherapy. Poor prognosis was associated with high expression levels of *KNSTRN* (Figures 8(c)–8(e)).

### 3.4. *KNSTRN* Causes Insensitivity to a Variety of Cancer Drugs

**Figure 9:**
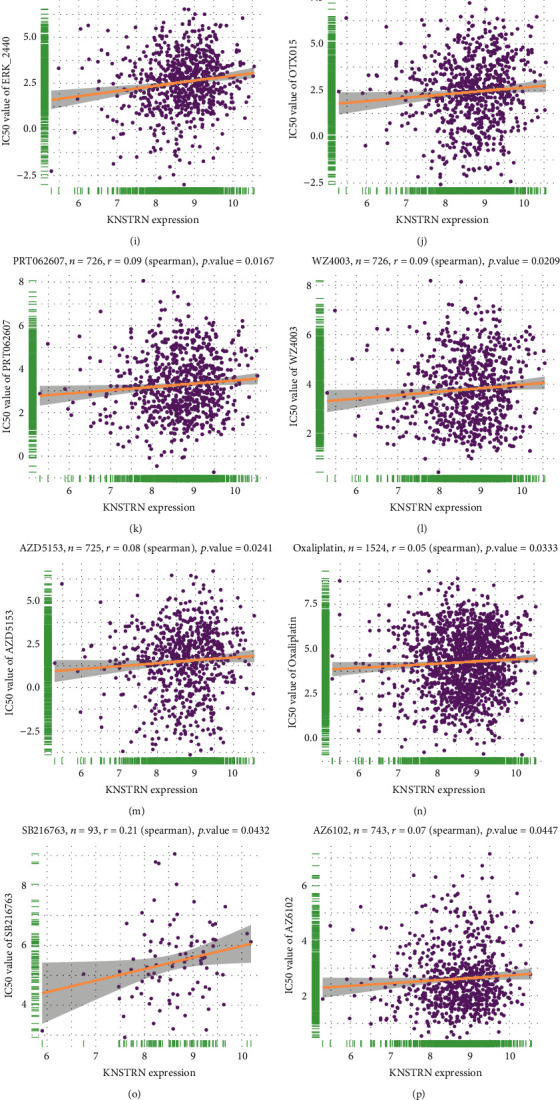
*KNSTRN* is related to the resistance of numerous anticancer drugs. (a–q) Analysis of the correlation of *KNSTRN* expression with the IC50 of some anticancer drugs using GDSC database. (s) The GSVA of *KNSTRN* in pan-cancer using the MsigDB database. ^∗^*p* < 0.05,  ^∗∗^*p* < 0.01,  ^∗∗∗^*p* < 0.001, and ^∗∗∗∗^*p* < 0.0001.

In addition to assessing the effect of *KNSTRN* on tumor immunotherapy outcomes, we further analyzed the relationship between *KNSTRN* expression and the IC50 of 192 antitumor drugs. *KNSTRN* expression upregulation was related to a higher IC50 in multiple antitumor drugs, such as Trametinib, Selumetinib, SCH772984, PD0325901, ERK_6604, VX-11e, Ulixertinib, Nutlin-3a (-), ERK_2440, OTX015, PRT062607, WZ4003, AZD5153, Oxaliplatin, SB216763, AZ6102, Doramapimod, and I-BET-762 (Figures 9(a)–9(r)). The reason for this decreased sensitivity caused by *KNSTRN* overexpression has not been clarified. GSVA (Figure 9(s)) revealed that the unfolded protein response (ER stress) was significantly positively correlated with *KNSTRN* expression, and ER stress has been reported by several studies to be an essential contributor to anti-tumor drug resistance. Therefore, ER stress may be one of the potential mechanisms through which *KNSTRN* contributes to anticancer drug resistance.

## 4. Discussion

To address uncontrolled tumor growth, metastasis [10, 11], and resistance to a variety of anticancer drugs, more effective tumor targets are needed in order to develop novel drugs and optimized treatment strategies [12]. Immunotherapy has played an increasingly crucial role in the treatment of solid malignant tumors. Nonetheless, some immunotherapy recipients experience little benefit because of the formation and persistence of an immunosuppressive microenvironment [13]. The structure and function of the TIME are altered by a decrease in the levels of immune-activating T cells, NK cells, and other effector immune cells and/or an increase in the levels of Tregs, immunosuppressive T cells, M2 macrophages, monocytes, and tumor-associated neutrophils. In addition, elevated expression of genes encoding immunosuppressive molecules promotes the formation of an immunosuppressive tumor microenvironment. For example, PD-L1 blockade, the most common immune checkpoint inhibitor, efficiently inhibits the growth of various malignant tumors. However, PD-L1 blockade has little therapeutic effect on the growth of recurrent glioblastoma, owing to the TIME, which features chemokines, chemokine receptors, immunosuppressive cytokines, suppressive immune cells, and extracellular vesicles [14]. Furthermore, despite the use of PD-1/PD-L1 blockade, which is the standard first-line treatment for non-small cell lung cancer, numerous patients with advanced cancer develop drug resistance and experience disease progression because of the immunosuppressive microenvironment [15]. However, in the local environment of hot tumors, the abundance of antitumor reactive cells may be positively correlated with the abundance of immune suppressive cells because of a feedback mechanism; antitumor inflammation usually promotes the recruitment or differentiation of cells dedicated to immunosuppression. This phenomenon indicates that the processes of immune activation and immunosuppression occur simultaneously [16]. This feedback mechanism also results in a positive correlation between the expression of immune-stimulatory genes and immune-inhibitory genes. Therefore, as our previous work has shown [17], molecules that have a significant impact on the TIME are likely to be positively associated with both immunosuppressive and immune-activating cells and genes. In general, manipulation of the TIME can serve as a useful strategy for combating resistance to immune checkpoint inhibitors.

In addition, despite the profound significance of immunotherapy, targeted therapy and chemotherapy remain important strategies for radical treatment, palliative care, and postoperative adjuvant therapy in several tumors, such as hematological malignancies, breast cancer, CRC, and lung cancer. In breast cancer, AKT-targeted therapy alleviates resistance to tumor immunotherapy and chemotherapy and controls tumor progression and immunosuppression [6]. In CRC, a combination of targeted therapy and chemotherapy can be used to induce tumor cell death. Panitumumab and the chemotherapeutic drug oxaliplatin are clinically effective against CRC [18]. Therefore, it is essential to investigate therapeutic sensitivity to targeted therapy and chemotherapeutic agents.

As a molecule mainly involved in cell cycle checkpoints, DNA replication, damage repair, and other biological processes [19–21], *KNSTRN* has rarely been studied in relation to tumor immunity. Only an analysis of lung adenocarcinoma identified a positive correlation between *KNSTRN* expression and the levels of Th2 and CD56^dim^ NK cells [7], which highlights the potential role of *KNSTRN* in tumor immunity. To address the gap in the literature regarding the role of *KNSTRN* in tumor immunity, we conducted a series of bioinformatics analyses. We first analyzed the RNA expression of *KNSTRN* in various cancer tissues and tumor cell lines. We also evaluated *KNSTRN* expression in cancer tissues, paired normal tissues, and tissues classified according to TNM stage. To better understand the expression of *KNSTRN* in tissues, we used the IHC data to assess the differential expression of *KNSTRN* between cancer tissues and paired normal tissues. We also analyzed immunofluorescence data to evaluate *KNSTRN* expression of distinct tumor cells at the cellular level.

The expression analysis results showed that the expression of *KNSTRN* in most tumor tissues was higher than that in normal tissues. High expression of *KNSTRN* was also associated with the advanced stages of multiple tumors. This is consistent with previous reports that *KNSTRN* is highly expressed in bladder cancer [7]. In various tumors, high expression of *KNSTRN* is usually associated with a worse prognosis. In esophageal cancer, *KNSTRN* expression is positively correlated with that of GSK-3*β*, which is associated with a poor prognosis [22].

To further explore the role of *KNSTRN* in the TIME, we analyzed the link between *KNSTRN* expression and levels of TIME-related components. The significant negative correlation between *KNSTRN* expression and immune score and infiltration score in multiple cancers indicated that *KNSTRN* has a negative effect on immune cell infiltration and immune activity in the TIME. Therefore, we further analyzed the correlation between *KNSTRN* expression and immune cell infiltration. *KNSTRN* expression was positively correlated with the infiltration of immunosuppressive cells, such as Tregs, neutrophils, and monocytes, and negatively correlated with the infiltration of immune-activating cells, such as NK cells and CD8 ^+^ effector T cells. Combined with data indicating a close association between *KNSTRN* and immune-related protein-encoding genes, these findings demonstrated the importance of *KNSTRN* in the TIME.

In addition, high *KNSTRN* expression was associated with a poor prognosis in patients receiving immunotherapy for certain tumors. *KNSTRN* was positively correlated with the IC50 of various anticancer drugs, suggesting that the overexpression of *KNSTRN* may promote resistance to anticancer drugs. To determine the mechanism by which *KNSTRN* induces drug insensitivity, we performed GSVA. The unfolded protein response, as one of several prominent enriched pathways, contributes to drug resistance. When addressing the clinical challenges of multidrug resistance and metastasis in breast cancer, some researchers have found that continuous ER stress and chemotherapy synergistically induce immunogenic cell death, which results in tumor suppression [23]. ER stress induces gastric cancer resistance to trastuzumab through the upregulation of the noncoding RNA miR-301a-3p [24].

In conclusion, high *KNSTRN* expression was identified in a variety of tumors. *KNSTRN* expression was closely associated with the TIME and with a poor prognosis in patients with tumors. *KNSTRN* may serve as a critical prognostic biomarker and potential therapeutic target that may affect sensitivity to immunotherapy and other anticancer treatments.

## 5. Conclusions

*KNSTRN* was highly expressed in various cancers. High *KNSTRN* expression was associated with a poor prognosis. *KNSTRN* affected the infiltration of multiple types of immune cells in the TIME and was closely associated with several immune-related genes in pan-cancer data. High expression of *KNSTRN* was associated with a poor prognosis in tumor patients receiving immunotherapy and was closely related to decreased sensitivity to other anticancer drugs. In summary, *KNSTRN* may serve as a prognostic biomarker and potential therapeutic target.

## Data Availability

The original contributions presented in the study are included in the article/supplementary material; further inquiries can be directed to the corresponding authors.

## Supplementary Materials

Supplementary Materials Figure S1: pan-cancer KNSTRN expression in tumor and paired normal tissues. (A-L) Evaluation of KNSTRN expression in tumor tissues and paired normal tissues from The Cancer Genome Atlas (TCGA) database. ^∗^*p* < 0.05,  ^∗∗^*p* < 0.01,  ^∗∗∗^*p* < 0.001, and^∗∗∗∗^*p* < 0.0001; ns: not significant. Figure S2: the role of KNSTRN in DFI, DSS, and PFI of cancer patients. (A–E) The role of KNSTRN in DFI of CESC, KIRP, LUSC, PAAD, and SARC patients. (F–O) The role of KNSTRN in DSS of ACC, BRCA, HNSC, KICH, KIRP, LGG, LIHC, LUAD, MESO, and PAAD patients. (P–Y) The role of KNSTRN in PFI of ACC, BRCA, CESC, GBM, HNSC, LIHC, LUAD, PAAD, and SARC patients. Figure S3: the correlation between the infiltration of NK resting cells, macrophages, Tregs, neutrophils, and KNSTRN of patients in pan-cancer. (A–F) The correlation between the infiltration of NK resting cells and KNSTRN of patients in multiple cancers using Published study. (G–N) The correlation between the infiltration of macrophages and KNSTRN of patients in multiple cancers using a published study. (O, P) The correlation between the infiltration of Tregs and KNSTRN of patients in multiple cancers using a published study. (Q–S) The correlation between the infiltration of neutrophils and KNSTRN of patients in multiple cancers using a published study.
