# Supplementary figures and images for: KNSTRN, a Poor Prognostic Biomarker, Affects the Tumor Immune Microenvironment and Immunotherapy Outcomes in Pan-Cancer
Source: Dis Markers. 2023 Feb 15;2023:6729717. doi: 10.1155/2023/6729717 (PMC9946745; doi:10.1155/2023/6729717)

**A**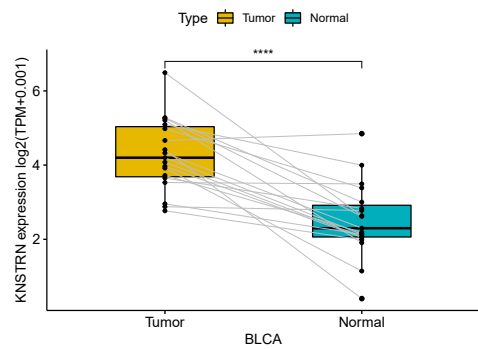**B**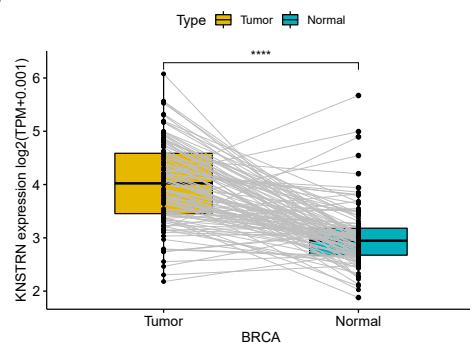**C**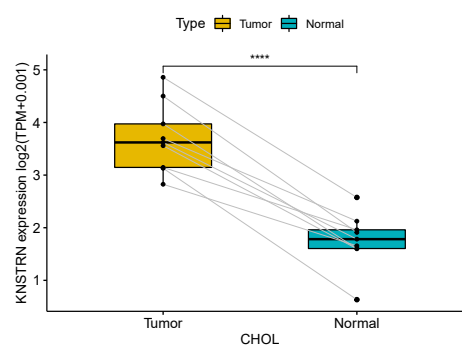**D**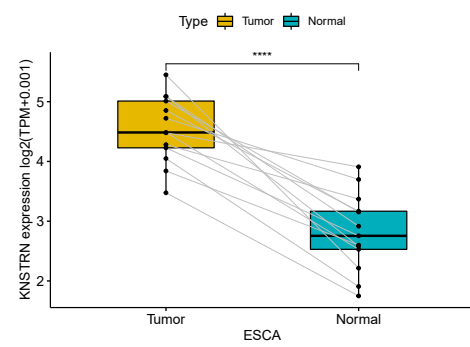**E**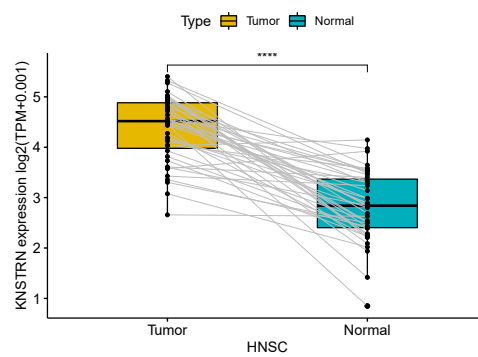**F**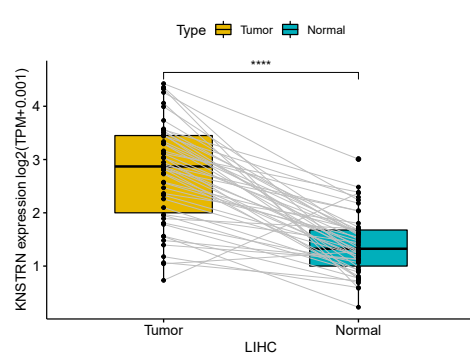**G**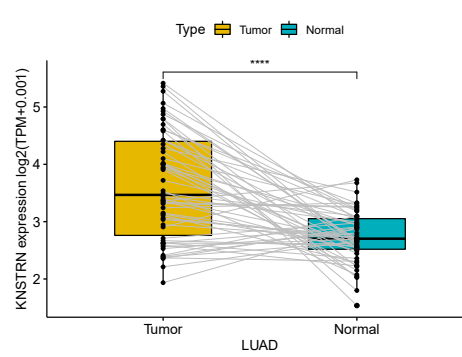**H**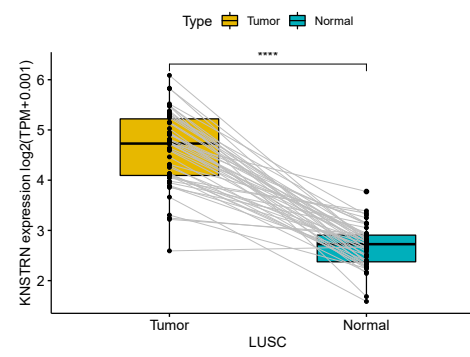**I**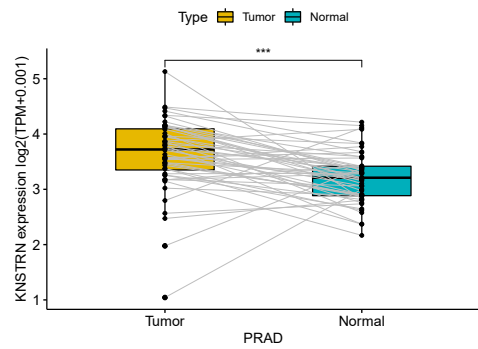**J**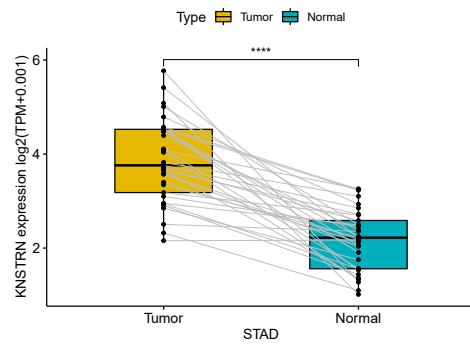**K**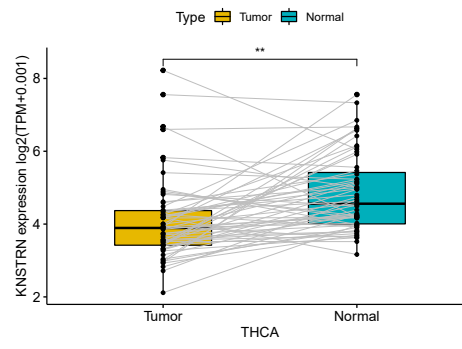**L**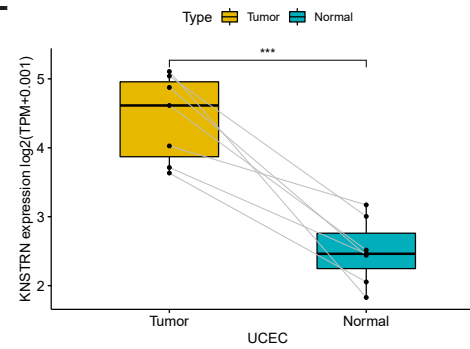

Supplement: Supplementary Materials — Figure S1: pan-cancer KNSTRN expression in tumor and paired normal tissues. (A-L) Evaluation of KNSTRN expression in tumor tissues and paired normal tissues from The Cancer Genome Atlas (TCGA) database. ∗p < 0.05, ∗∗p < 0.01, ∗∗∗p < 0.001, and∗∗∗∗p < 0.0001; ns: not significant. Figure S2: the role of KNSTRN in DFI, DSS, and PFI of cancer patients. (A–E) The role of KNSTRN in DFI of CESC, KIRP, LUSC, PAAD, and SARC patients. (F–O) The role of KNSTRN in DSS of ACC, BRCA, HNSC, KICH, KIRP, LGG, LIHC, LUAD, MESO, and PAAD patients. (P–Y) The role of KNSTRN in PFI of ACC, BRCA, CESC, GBM, HNSC, LIHC, LUAD, PAAD, and SARC patients. Figure S3: the correlation between the infiltration of NK resting cells, macrophages, Tregs, neutrophils, and KNSTRN of patients in pan-cancer. (A–F) The correlation between the infiltration of NK resting cells and KNSTRN of patients in multiple cancers using Published study. (G–N) The correlation between the infiltration of macrophages and KNSTRN of patients in multiple cancers using a published study. (O, P) The correlation between the infiltration of Tregs and KNSTRN of patients in multiple cancers using a published study. (Q–S) The correlation between the infiltration of neutrophils and KNSTRN of patients in multiple cancers using a published study. [file 6729717.f1.zip › Figure S1 (2).pdf]

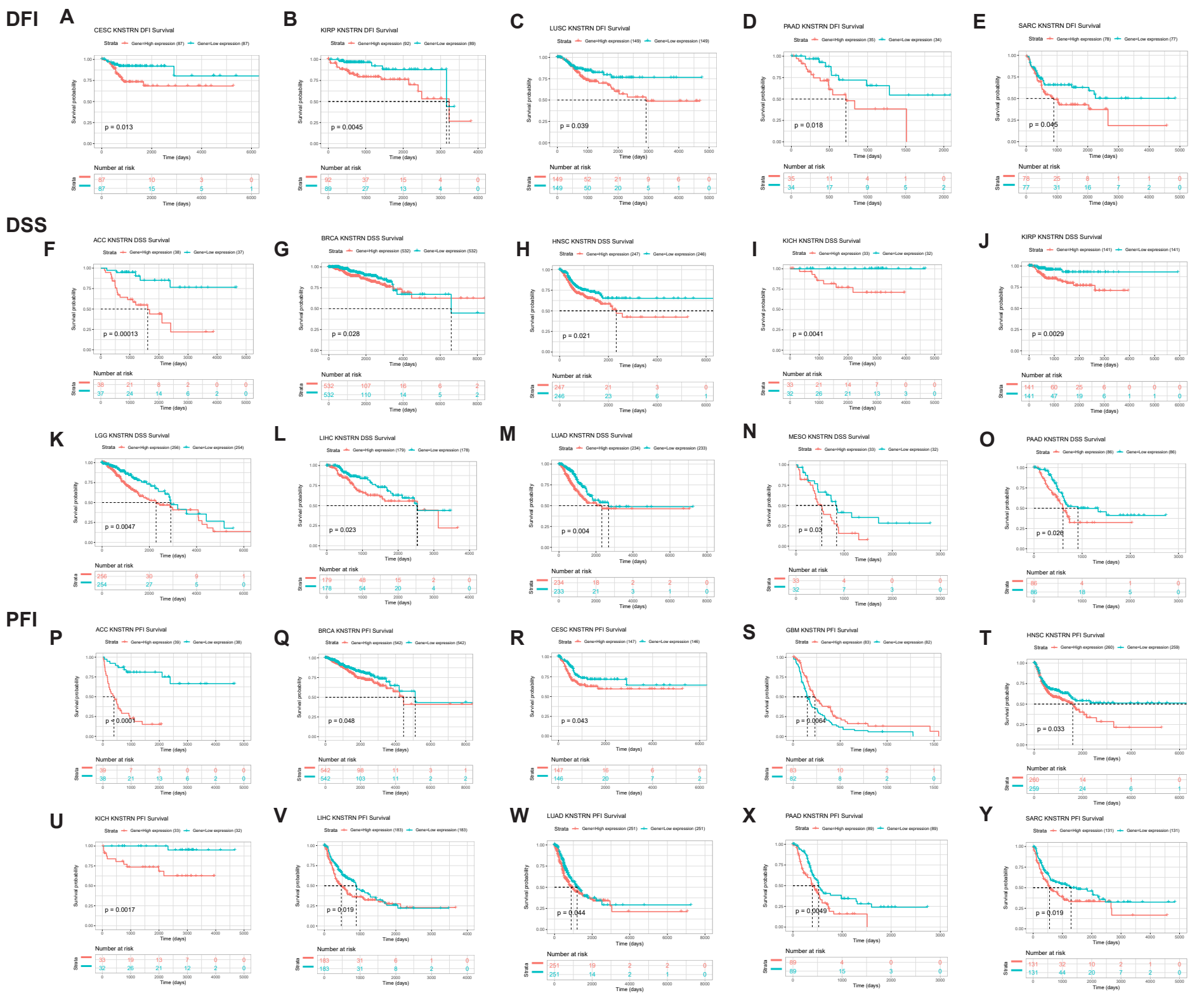

Supplement: Supplementary Materials — Figure S1: pan-cancer KNSTRN expression in tumor and paired normal tissues. (A-L) Evaluation of KNSTRN expression in tumor tissues and paired normal tissues from The Cancer Genome Atlas (TCGA) database. ∗p < 0.05, ∗∗p < 0.01, ∗∗∗p < 0.001, and∗∗∗∗p < 0.0001; ns: not significant. Figure S2: the role of KNSTRN in DFI, DSS, and PFI of cancer patients. (A–E) The role of KNSTRN in DFI of CESC, KIRP, LUSC, PAAD, and SARC patients. (F–O) The role of KNSTRN in DSS of ACC, BRCA, HNSC, KICH, KIRP, LGG, LIHC, LUAD, MESO, and PAAD patients. (P–Y) The role of KNSTRN in PFI of ACC, BRCA, CESC, GBM, HNSC, LIHC, LUAD, PAAD, and SARC patients. Figure S3: the correlation between the infiltration of NK resting cells, macrophages, Tregs, neutrophils, and KNSTRN of patients in pan-cancer. (A–F) The correlation between the infiltration of NK resting cells and KNSTRN of patients in multiple cancers using Published study. (G–N) The correlation between the infiltration of macrophages and KNSTRN of patients in multiple cancers using a published study. (O, P) The correlation between the infiltration of Tregs and KNSTRN of patients in multiple cancers using a published study. (Q–S) The correlation between the infiltration of neutrophils and KNSTRN of patients in multiple cancers using a published study. [file 6729717.f1.zip › Figure S2 (2).pdf]

NK resting cells

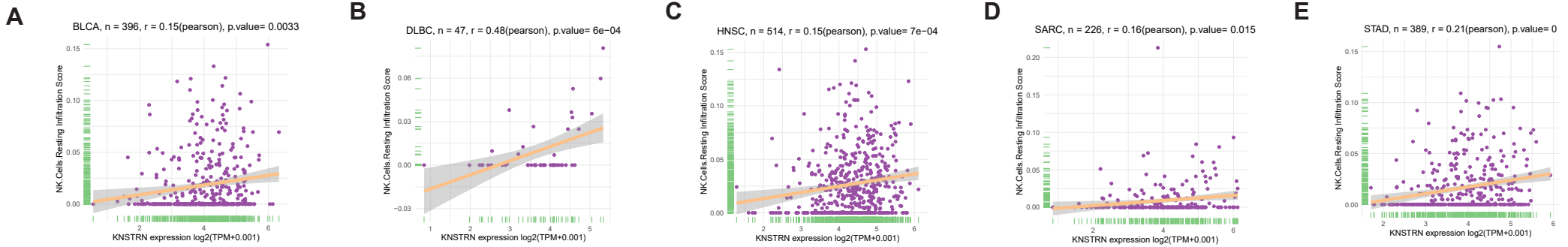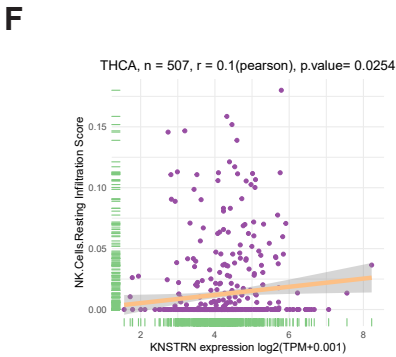

Macrophage

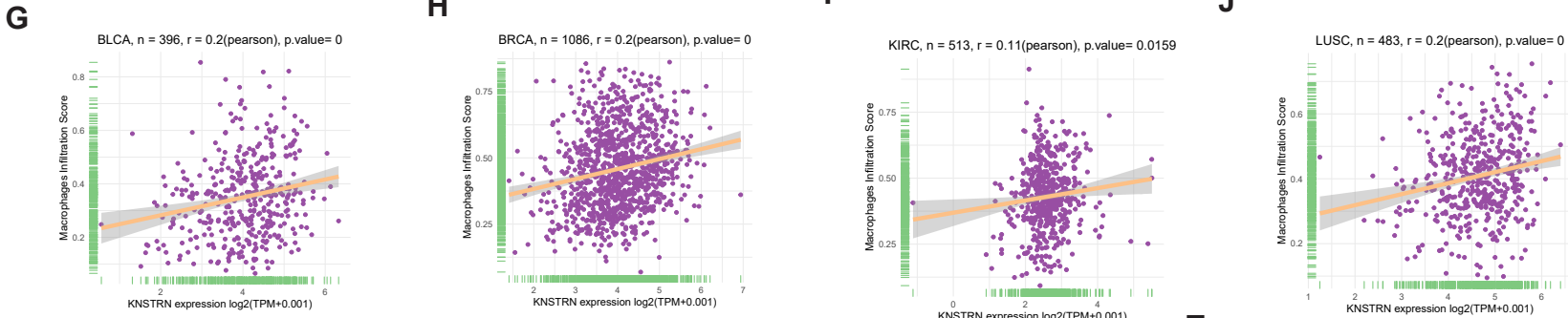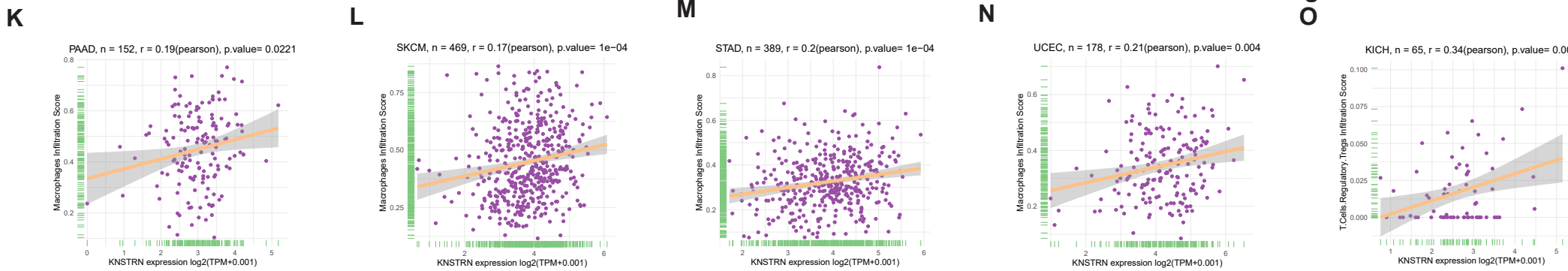

Neutrophils

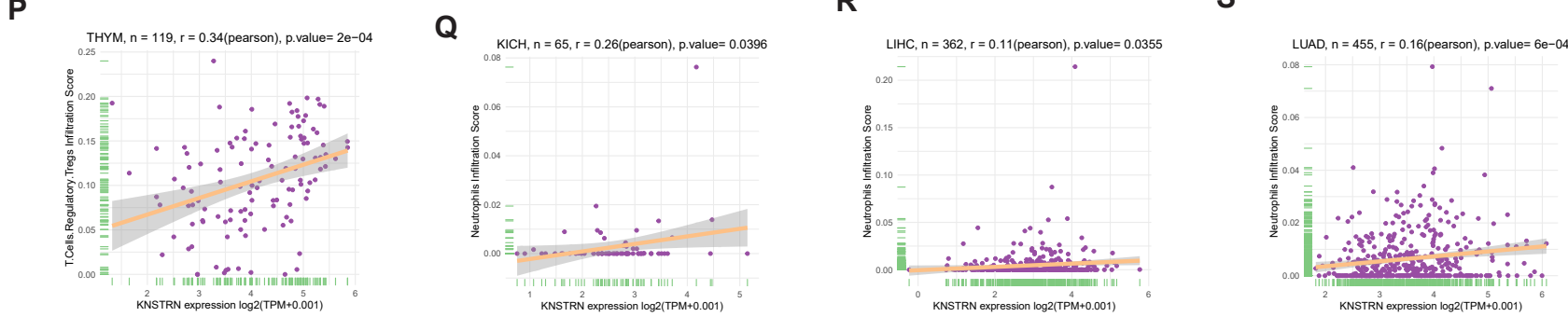

Supplement: Supplementary Materials — Figure S1: pan-cancer KNSTRN expression in tumor and paired normal tissues. (A-L) Evaluation of KNSTRN expression in tumor tissues and paired normal tissues from The Cancer Genome Atlas (TCGA) database. ∗p < 0.05, ∗∗p < 0.01, ∗∗∗p < 0.001, and∗∗∗∗p < 0.0001; ns: not significant. Figure S2: the role of KNSTRN in DFI, DSS, and PFI of cancer patients. (A–E) The role of KNSTRN in DFI of CESC, KIRP, LUSC, PAAD, and SARC patients. (F–O) The role of KNSTRN in DSS of ACC, BRCA, HNSC, KICH, KIRP, LGG, LIHC, LUAD, MESO, and PAAD patients. (P–Y) The role of KNSTRN in PFI of ACC, BRCA, CESC, GBM, HNSC, LIHC, LUAD, PAAD, and SARC patients. Figure S3: the correlation between the infiltration of NK resting cells, macrophages, Tregs, neutrophils, and KNSTRN of patients in pan-cancer. (A–F) The correlation between the infiltration of NK resting cells and KNSTRN of patients in multiple cancers using Published study. (G–N) The correlation between the infiltration of macrophages and KNSTRN of patients in multiple cancers using a published study. (O, P) The correlation between the infiltration of Tregs and KNSTRN of patients in multiple cancers using a published study. (Q–S) The correlation between the infiltration of neutrophils and KNSTRN of patients in multiple cancers using a published study. [file 6729717.f1.zip › Figure S3 (2).pdf]
